# Global prevalence and associated factors of intestinal parasitic infections among institutionalized populations: a systematic review and meta-analysis

**DOI:** 10.1186/s12879-025-12228-z

**Published:** 2025-11-24

**Authors:** Albert Abaka-Yawson, Daniel Sai Squire, Ibrahim Issah, Kenneth Ablordey, Emmanuel Ativi, Serwaa Akoto Bawua, John Arko-Mensah

**Affiliations:** 1 Department of Biological, Environmental and Occupational Health Sciences, School of Public Health, University of Ghana, Legon, Accra Ghana; 2 Department of Medical Laboratory Science, School of Allied Health Sciences, University of Health and Allied Sciences, Ho, Ghana; 3 West Africa Center for Global Environmental, & Occupational Health, College of Health Sciences, University of Ghana, Legon, Accra Ghana

**Keywords:** IPI, Associated factors, Institutionalized populations, Worldwide, Meta-analysis

## Abstract

**Background:**

Various studies have been conducted on intestinal parasitic infections among different institutionalized populations with varying outcomes. We aimed at conducting a systematic review and meta-analysis on the prevalence and associated factors of intestinal parasitic infections (IPIs) among institutionalized populations.

**Methods:**

Articles published from January 2000 to October 2025 were retrieved from online sources such as Medline/ PubMed, Google Scholar and Scopus. Two authors (AAY and EA) independently extracted and reviewed all relevant data using a standardized Microsoft Excel data extraction form. Funnel plots and Egger’s tests were used to assess publication bias, followed by trim-and-fill analysis. A p-value < 0.05 indicates statistical significance. The pooled estimates and associated factors were assessed with a random-effects model using Stata/ SE version 17.0.

**Results:**

The pooled sample size for the study was 35,716 drawn from 59 journal articles. These articles included 21 (35.6%) from refugees, 17 (28.8%) from prison inmates, 13 (22.0%) mentally retarded institutions, 3 (5.1%) elderly nursing homes, 2 (3.4%) rehabilitation centers, 1 (1.7%) long-term care facilities and 2 (3.4%) military camps. The overall prevalence of IPIs among institutionalized populations was found to be 34.0% (95% CI: 29.0%, 39.0%). Based on type of institution, rehabilitation centers had the highest prevalence of IPIs with 57.0% (95% CI: 39.0%, 76.0%). Additionally, based on continent, Australia presented with the highest prevalence of IPIs with 65.8% (95% CI: 57.2, 74.4). Again, this review found *Blastocystis hominis* as the most prevalent protozoan prevalent among institutionalized populations at 18.6% (95% CI: 13.2–24.1), and *Ascaris lumbricoides* as the most prevalent helminth 5.0% (95% CI: 3.9–6.1). Studies from mentally retarded institutions and prisons showed that trimmed fingernails were associated with reduced odds of IPIs (pooled OR: 0.53, 95%CI: 0.40–3.44, *p* < 0.010).

**Conclusion:**

The pooled prevalence showed the presence of IPIs in about a third (34%) of institutionalized populations, presenting a major public health concern. Additionally, untrimmed fingernails in various institutions seemed to predispose participants to IPIs. It is therefore recommended that health professionals conduct periodic screening and treatment for IPIs. Lastly, institutional leaders, particularly those of psychiatric and prisons facilities should consider offering nail care services to control the transmission of IPIs.

**Supplementary Information:**

The online version contains supplementary material available at 10.1186/s12879-025-12228-z.

## Introduction

Globally, intestinal parasitic infections (IPIs) present a significant public health concern, with 450 million currently living with the infections [1]. They are caused by protozoans and helminths which are mainly found in places with limited access to potable water, as well as poor sanitation and hygiene [2]. These parasites are spread from person-to-person via faeco-oral route through contact with contaminated food or water [2]. Generally, the infective stage of protozoans and helminths are ova, cysts and larva [3], which could be determined notably by microscopy, by serology, polymerase chain reaction (PCR) test among others.

Institutionalized populations refer to persons living in groups of an institutional facility, usually confined or semi-confined [4]. These include prisons, mental health/ psychiatric homes, orphanages, nursing homes, refugee camps among others. These facilities are usually characterized by overcrowding, poor sanitation, compromised health and limited access to quality health [5, 6], thus making them vulnerable and susceptible to IPIs.

Various studies have reported different prevalence rates of IPIs among institutionalized populations, with some prison inmates having prevalence as high as 72.7% [7], 70.0% among refugees [8], and 54.76% in psychiatric homes [9]. A recent systematic review and meta-analysis reported a pooled prevalence rate of 30.12% among prison inmates [10]. Additionally, predominant protozoans and helminths among these groups have been determined to be *Entamoeba histolytica* and *Ascaris lumbricoides*, which are known to cause malnutrition, anaemia and gastrointestinal disturbances [11–13]. This makes IPIs an important public health concern.

Additionally, the association between factors such as handwashing practices [11, 12, 14, 15], fingernail trimming practices [11, 14, 16], duration of stay [11, 12, 16], level of education [11, 14], gender [9, 11, 17–21] and IPIs have been previously assessed among institutionalized populations with divergent findings with some studies pointing to its significance whilst others do not.

This frustrates the effort of the World Health Organization Neglected Tropical Diseases Elimination Strategy 2030, which aims at providing interventions based on data quality [22]. This study therefore seeks to address this gap with a systematic review and meta-analysis to pool together available data to draw a meaningful conclusion, for management and control of these neglected infections.

## Methods

This systematic review and meta-analysis was reported following the Preferred Reporting Items for Systematic Reviews and Meta-Analyses (PRISMA) guideline. The protocol for the development of this systematic review and meta-analysis was registered on the International Prospective Register of Systematic Reviews (PROSPERO) under reference number (CRD420250551548).

### Eligibility criteria

#### Inclusion criteria

Articles were included in this study if they met the following criteria: (1) studies focusing on institutionalized populations and employing, observational epidemiological designs, published in peer-reviewed journals and reporting prevalence of IPIs; (2) studies published online between January 1, 2000 and October 8, 2025; (3) availability of the abstract or full-text in English; and (4) evidence of at least one type of intestinal parasite using parasitological methods (microscopy, PCR or serological methods).

#### Exclusion criteria

Articles were excluded based on the following criteria: (1) articles in languages other than English; (2) case reports, reviews and studies without full-text access despite at least two attempts to contact the primary authors were excluded, as full access was necessary for quality assessment.

### Information sources and search strategy

Two authors (AAY and EA) independently retrieved peer-reviewed articles that reported on IPIs among institutionalized populations from Google Scholar, Medline/PubMed and Scopus. The PubMed search strategy included the tailored terms (“Intestinal Diseases, Parasitic“[Mesh] OR gastrointestinal parasitic infection) AND (((“Prisoners“[Mesh]) OR (“Correctional Facilities“[Mesh])) OR (“Hospitals, Psychiatric“[Mesh]) OR (“Refugee Camps“[Mesh] OR “Refugees“[Mesh]) OR (“Nursing Homes“[Mesh]) OR (“Homes for the Aged“[Mesh]) OR (“Rehabilitation Centers“[Mesh])) AND (“risk factors“[MeSH] OR associated factors[tiab]). Similar terms were adapted for other databases. Reference lists of included studies were manually screened for additional articles.

### Selection process

**Fig. 1:**
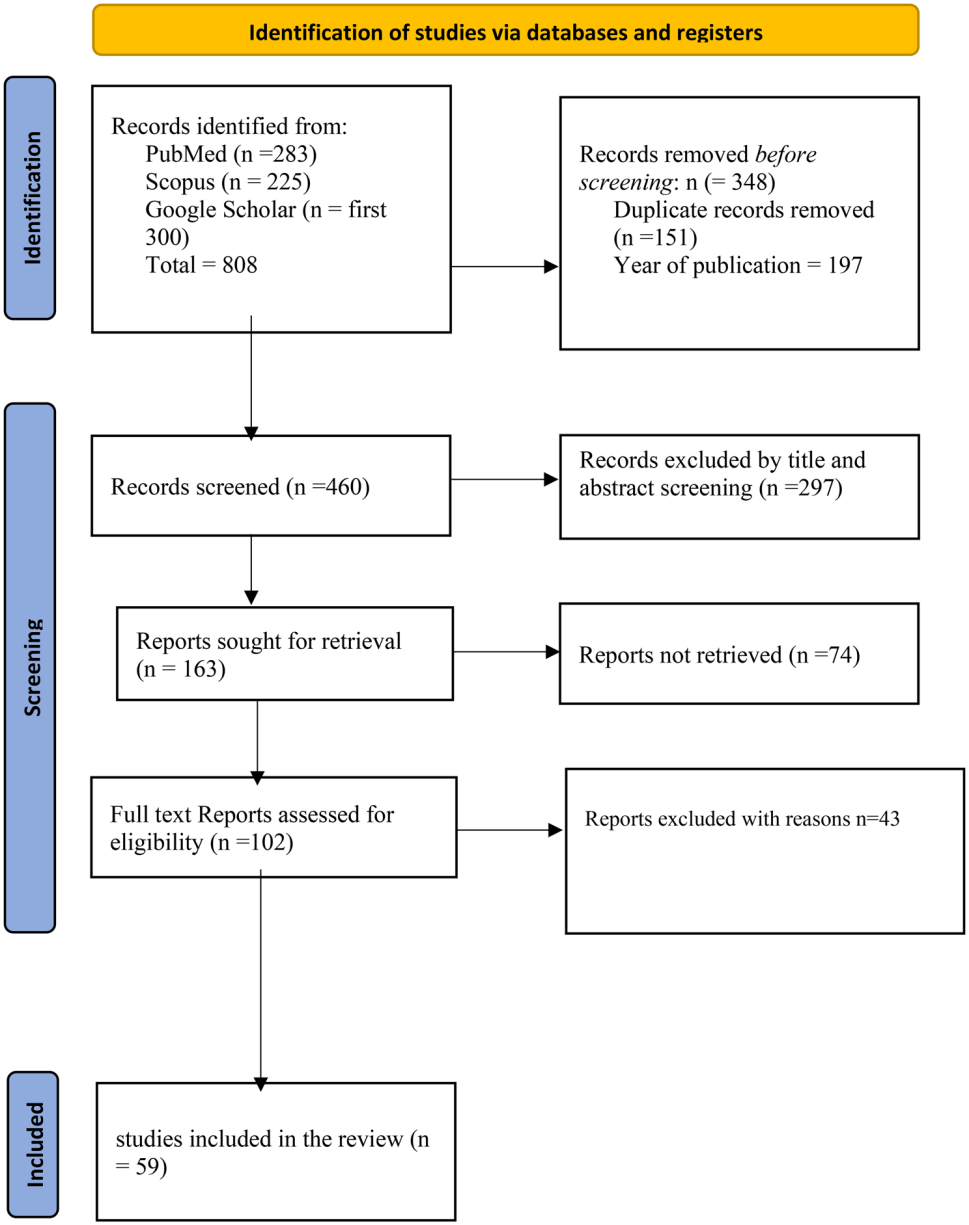
PRISMA flow chart on selection of articles for this study

All retrieved records were imported into EndNote version 21.3 for reference management, where duplicate entries were identified and removed using the software’s duplicate detection feature. The remaining studies underwent a three-stage screening process consisting of title, abstract, and full-text screening, conducted independently by two reviewers (AAY and EA) based on the predefined exclusion and inclusion criteria. Any discrepancies were resolved through discussion or consultation with a third reviewer (KA). The screening process was managed using Microsoft excel 365 to ensure accuracy and consistency, and the overall selection process was summarized using a PRISMA flow diagram (Fig. 1).

### Data extraction

Two authors (AAY and EA) independently extracted all data using a uniform extraction format designed in Microsoft Excel 365. The format included first authors (years) and title of paper, study period, study design, sample size, prevalence of IPI, type of sample, diagnosis, country and population type. Any disagreements between the authors during the extraction process were resolved by mutual agreement or with the help of a third author (DSS).

### Quality assessment

The quality of the studies was assessed using the Newcastle Ottawa Scale (NOS) for cross-sectional and cohort studies as described by Elyasi et al. [23]. Two reviewers independently (AAY and KA) assessed the risk of bias studies based on the tool above, and EA resolved any discrepancies. Three dimensions of quality were assessed: Selection (three items), Comparability (two items), Exposure (one item) and Outcome (one item). The NOS score ranges from 0 (lowest grade) to 10 (highest grade). Studies with NOS scores greater than 5 were graded as high quality as seen in Appendix 1.

### Outcome measurements

This systematic review and meta-analysis had two objectives. The first objective was to determine the reported prevalence of IPI by microscopy, PCR or serology. The prevalence was calculated by dividing the number of infected individuals with at least one IPI by the total number of individuals who have been included in the study (sample size) multiplied by 100. The second objective of the study was to identify the factors associated with the IPI. The odds ratios of associated factors were calculated using the two by-two tables with binary outcomes gender (male/female), handwashing after using toilet (yes/ no), handwashing before meals (yes/ no), methods of handwashing (water only/ soap and water), literacy (educated/ not educated), trimmed fingernails (trimmed/untrimmed), duration of imprisonment (less than 3 years/ 3 years and above).

### Meta analysis

Meta analyses were carried out using Stata software version 17.0, (Stata Corp, Texas, USA). The inverse of variance (I^2^) statistic was used to assess the level of inconsistency between the included research. The I^2^ statistic measures the proportion of variation between studies that is due to heterogeneity rather than chance. The I^2^ values were interpreted using specific thresholds: 0–25%, 25–50%, 50–75%, with values greater than 75% indicating inconsequential, then low, moderate, and high heterogeneity respectively. A random-effect model was used for moderate and high heterogeneity.

To account for significant heterogeneity across studies, we performed a random effects meta-analysis and reported results as pooled prevalence and 95% confidence intervals (95% CI). Heterogeneity between studies was identified using meta-regression and subgroup analysis. To ensure uniformity, a sensitivity analysis was carried out by removing individual research. Funnel plots and Egger’s tests were used to assess publication bias, followed by trim-and-fill analysis. A P-value < 0.05 indicates statistical significance.

## Results

### Study selection

The study included articles published between 1 January 2000 and 8 October 2025. The PRISMA flow diagram illustrates the selection process for studies included in this review. A total of 808 records were identified through database searches-PubMed (*n* = 283), Scopus (*n* = 225), and the first 300 results from Google Scholar. Before screening, 298 records were removed, comprising 151 duplicates and 197 records excluded based on the year of publication. This left 460 records for title and abstract screening, from which 297 were excluded due to irrelevance. Of the remaining 163 reports sought for retrieval, 61 could not be accessed. Ultimately, 59 studies met the inclusion criteria and were included in the final review (Fig. 1). A detailed PRISMA Checklist is presented in Appendix 2.

### Study characteristics

A total of 59 studies were included in this review, spanning various countries and targeting vulnerable and institutionalized populations such as prison inmates, refugees, individuals in psychiatric or mentally retarded institutions, military personnel, the elderly in nursing homes, and residents of long-term care or rehabilitation centers. Most studies employed cross-sectional designs with sample sizes ranging from 12 to 17,911 individuals. Stool samples were the primary diagnostic material, and microscopy was the most common diagnostic method, although some studies also used serology, ELISA, PCR, or fluorescent antibody testing. Reported prevalence rates of IPIs varied widely, from as low as 2.5% among military personnel to as high as 91.89% in elderly individuals with cognitive impairment in Malaysia (Table 1).

**Table 1:** Characteristics of included studies

Authors (Year)	Study Design (Sample Size)	Country	Population Type	Prevalence	Diagnosis Method
Abaka-Yawson et al. (2024) [11]	Cross-sectional study (461)	Ghana	Prisons inmates	38.2%	Microscopy
Agmas et al. (2021) [14]	Cross-sectional study (432)	Ethiopia	Mentally retarded institution	38.90%	Microscopy
Ameya et al. (2019) [24]	Cross-sectional study (320)	Ethiopia	Prisons inmates	48.10%	Microscopy
Amit et al. (2016) [25]	cross sectional study (114)	India	Prisons inmates	7.90%	Microscopy
Angal et al. (2015) [26]	Cross-sectional study (294)	Malaysia	Prisons inmates	26.50%	Microscopy and Serology
Board & Suzuki (2015) [27]	Cross-sectional study (9860)	USA	Refugees	25.30%	Microscopy
Caruana et al. (2006) [28]	Cross-sectional study (127)	Australia	Refugees	65.8%	Serology and ELISA
Chandrasena et al. (2010) [29]	cross sectional study (145)	Sri Lanka	Mentally retarded institution	33%	Microscopy
Chang et al. (2013) [30]	Retrospective cohort study (1376)	USA	Refugees	12.3%	Microscopy
Cheng & Wang (2018) [31]	Cross-sectional study (736)	Taiwan	Refugees	8.40%	Microscopy
Chernet et al. (2018) [32]	Cross-sectional study (151)	Switzerland	Refugees	10.3%	Microscopy and ELISA
Curval et al. (2017) [33]	Cross-sectional study (510)	Brazil	Prisons inmates	20.20%	Microscopy
Demirel & Dinc et al. (2022) [34]	Retrospective Observational study (17911)	Turkey	Refugees	3.60%	Microscopy
De Vetten et al. (2017) [17]	Retrospective study (1390)	Norway	Refugees	29.71%	Microscopy and direct fluorescent antibody testing
Duedu et al. (2015) [35]	Cross-sectional study (111)	Ghana	Mentally retarded institution	13.50%	Microscopy
Eze et al. (2019) [21]	Cross-sectional study (203)	Nigeria	Mentally retarded institution	38.40%	Microscopy
Frickmann et al. (2013) [36]	Retrospective cohort study (1122)	German	Military camp	2.50%	PCR
Fujishima et al. (2010) [37]	Case Series (76)	Japan	Rehabilitation center	67.10%	Microscopy, Serology
Garg et al. (2005) [38]	Cross-sectional study (772)	USA	Refugees	14%	Microscopy
Gatti et al. (2000) [39]	Cross-sectional study (550)	Italy	Mentally retarded institution	23%	Microscopy, Serology
Geltman et al. (2003) [40]	Prospective descriptive (1254)	USA	Refugees	56%	Microscopy
Girotto et al. (2013) [41]	Cross-sectional study (293)	Brazil	Elderly nursing home	9.50%	Microscopy
Haq (2015) [42]	Cross-sectional (687)	Pakistan	Refugees	55.40%	Microscopy
Khayar et al. (2024) [43]	Retrospective study (372)	Jordan	Refugees	63.7%	Microscopy
Korzeniewski et al. (2024) [44]	Cross-sectional study (221)	Poland	Military Camp	12.60%	Microscopy
Korzeniewski et al. (2024) [45]	Cross-sectional study (127)	Ukraine	Refugees	22.50%	Microscopy
Kuete et al. (2019) [46]	Cross-sectional study (374)	Cameroon	Prisons inmates	39.30%	Microscopy
Lee et al. (2018) [8]	Case series (20)	South Korea	Refugees	70.00%	Microscopy
Leung et al. (2019) [47]	Retrospective descriptive study (12)	Canada	Prisons inmates	7.80%	Microscopy
Malla et al. (2017) [48]	Descriptive study (393)	Cameroon	Prisons inmates	6.87%	Microscopy
Mamo (2014) [7]	Cross-sectional study (121)	Ethiopia	Prisons inmates	72.70%	Microscopy
Mardu et al. (2019) [13]	Cross-sectional study (270)	Ethiopia	Prisons inmates	40%	Microscopy
Mardu et al. (2019) [49]	Cross-sectional study (291)	Ethiopia	Prisons inmates	42.60%	Microscopy
Mbaawuaga et al. (2024) [50]	Cross-sectional study (400)	Nigeria	Refugees	28.8%	Microscopy
Miller et al. (2000) [51]	Cross-sectional study (390)	USA	Refugees	38%	Microscopy
Mitchell et al. (2018) [52]	Prospective Cohort study (3419)	USA	Refugees	72.70%	Microscopy, Serology and PCR
Mohammadi-Meskin et al. (2019) [9]	Cross-sectional study (163)	Iran	Mentally retarded institution	54.76%	Microscopy
Munoz-Antoli et al. (2025) [53]	Cross-sectional study (471)	Spain	Prisons inmates	7.90%	PCR
Munoz-Antoli et al. (2023) [19]	Cross-sectional study (528)	Spain	Prisons inmates	15.70%	Microscopy
Mussema et al. (2024) [12]	Cross-sectional study (420)	Ethiopia	Prisons inmates	39.20%	Microscopy
Naves & Costa-Cruz (2013) [54]	Cross-sectional study (200)	Brazil	Elderly nursing home	8.00%	Microscopy
Oduma & Ukpen (2024) [55]	Cross-sectional study (85)	Nigeria	Mentally retarded institution	27.10%	Microscopy
Nyundo et al. (2017) [56]	Cross-sectional study (233)	Tanzania	Mentally retarded institution	12.49%	Microscopy
Otu-Bassey et al. (2019) [57]	Cross-sectional study (126)	Nigeria	Mentally retarded institution	49.20%	Microscopy
Peters et al. (2021) [58]	Retrospective descriptive study (4695)	USA	Refugees	45.73%	Microscopy
Richert et al. (2024) [20]	Cross-sectional study (25)	Poland	Refugees	20.00%	Microscopy
Rivera et al. (2006) [59]	Cross-sectional study (113)	Philippines	Mentally retarded institution	70.80%	Microscopy, Serology and PCR
Ferrer-Rodriguez & Kozek (2011) [60]	Cross-sectional study (111)	Puerto Rico	Mentally retarded institution	52.3%	Microscopy
Rop et al. (2016) [61]	Cross sectional study (384)	Kenya	Prisons inmates	24.70%	Microscopy
Saeidina et al. (2016) [62]	Cross-sectional study (173)	Iran	Mentally retarded institution	29.50%	Microscopy
Seybolt et al. (2006) [63]	Retrospective chart review (265)	USA	Refugees	29.00%	microscopy and serology
Shehata & Hassanein (2015) [64]	Cross-sectional study (200)	Egypt	Mentally retarded institution	42.59%	Microscopy
Shokri et al. (2012) [65]	Cross-sectional study (133)	Iran	Rehabilitation center	48.50%	Microscopy
Shrestha et al. (2019) [18]	Cross-sectional study (400)	Nepal	Prisons inmates	6.00%	Microscopy
Su et al. (2009) [66]	Cross-sectional study (713)	Taiwan	long-term care facilities	5.30%	Microscopy
Fontanelli Sulekova et al. (2019) [67]	Cross-sectional study (391)	Italy	Refugees	20.60%	Microscopy
Tamomh et al. (2025) [15]	Cross-sectional study (508)	Sudan	Refugees	33.90%	Microscopy
Terefe et al. (2019) [16]	Cross-sectional study (234)	Ethiopia	Prisons inmates	47.40%	Microscopy
Zamari et al. (2023) [68]	Cross-sectional study (37)	Malaysia	Elderly nursing home	91.89%	Microscopy

### Prevalence of intestinal parasitic infections among various institutionalized populations

**Fig. 2:**
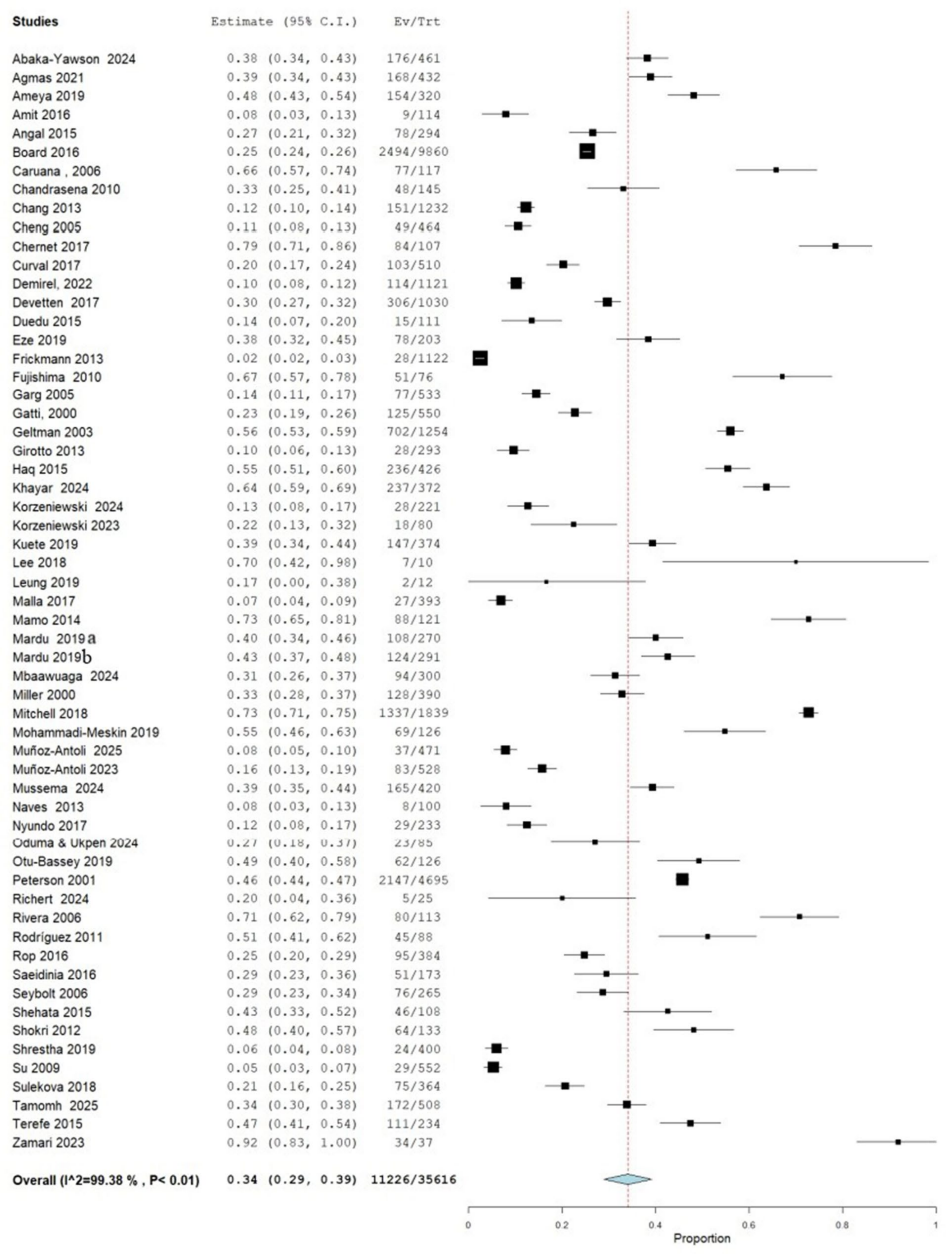
Prevalence of IPIs among people living in institutional facilities

As presented in Fig. 2, the forest plot depicts the prevalence estimates and corresponding 95% confidence intervals for IPIs across various studies conducted in different institutionalized population groups and settings. The overall prevalence estimate, represented by the diamond at the bottom, is 0.34 (95% CI: 0.29, 0.39), indicating that approximately 34.0% of the combined study population had IPIs. The plot reveals a considerable variation in the prevalence rates across different studies, ranging from as low as 2.5% in a study on German soldiers [36] to as high as 91.89% in a study on a geriatric community with cognitive impairment in Malaysia [68].

### Meta-analysis of the prevalence of range of intestinal parasites from included studies among institutionalized populations

The review revealed *Blastocystis hominis* as the most prevalent organism at 18.6% (95% CI: 13.2–24.1). Other organisms with high prevalence rates included *Entamoeba. coli* (4.9%, 95% CI: 3.8-6.0), *Endolimax nana* (7.4%, 95% CI: 4.4–10.4), *Giardia spp.* (7.5%, 95% CI: 6.0-9.1) and *Entamoeba histolytica/dispar/mashkovskii* (6.7%, 95% CI: 5.6–7.7). *Ascaris lumbricoides* and Hookworm showed moderate prevalence rates of 5.0% (95% CI: 3.9–6.1) and 3.6% (95% CI: 2.7–4.5) respectively. Additionally, other organisms with prevalence rates include *Chilomastix mesnili* (1.9%, 95% CI: 0.5–3.4), *Hymenolepis nana* (1.7%, 95% CI: 1.0-2.5), *Enterobius vermicularis* (1.1%, 95% CI: 0.5–1.6). Organisms such as *S. mansoni*, *Cryptosporidium parvum*, *Clornorchis spp.* and *Taenia spp*, demonstrated prevalence rates below 1%. See Table 2.

**Table 2:** Meta-analysis of the prevalence of various intestinal parasites from included studies among institutionalized populations

Intestinal Parasite	Studies reviewed	Prevalence % (95% CI)	I^2^ (%)	*P*-value
**Protozoa**				
*Blastocystis hominis*	20	18.6 (13.2–24.1)	99.32	< 0.001
*Entamoeba histolytica/dispar/mashkovskii*	29	6.7 (5.6–7.7)	96.97	< 0.001
*Giardia species*	37	7.5 (6.0-9.1)	97.44	< 0.001
*Cryptosporidium parvum*	8	0.8 (0.3–1.3)	61.7	0.011
*Entamoeba. Coli*	21	4.9 (3.8-6.0)	94.37	< 0.001
*Dientamoeba fragilis*	5	4.7 (1.8–7.6)	92.31	< 0.001
*Chilomastix mesnili*	5	1.9 (0.5–3.4)	92.68	< 0.001
*Endolimax nana*	13	7.4 (4.4–10.4)	98.34	< 0.001
**Helminths**				
*Trichuris trichiura*	26	3.3 (2.5–4.1)	96.19	< 0.001
*Ascaris lumbricoides*	19	5.0 (3.9–6.1)	95.46	< 0.001
*Schistosoma mansoni*	14	0.7 (0.4–1.1)	77.83	< 0.001
Hookworm	18	3.6 (2.7–4.5)	92.54	< 0.001
*Strongyloides stercoralis*	18	1.5 (1.0–2.1)	91.65	< 0.001
*Clornorchis spp*	3	0.2 (0.2–0.6)	34.7	0.216
*Taenia spp*	9	0.7 (0.3–1.2)	71.62	< 0.001
*Enterobius vermicularis*	12	1.1 (0.5–1.6)	94.21	< 0.001
*Hymenolepsis nana*	15	1.7 (1.0-2.5)	93.2	< 0.001

### Sub-group analysis of the prevalence of IPIs based on continent

**Fig. 3:**
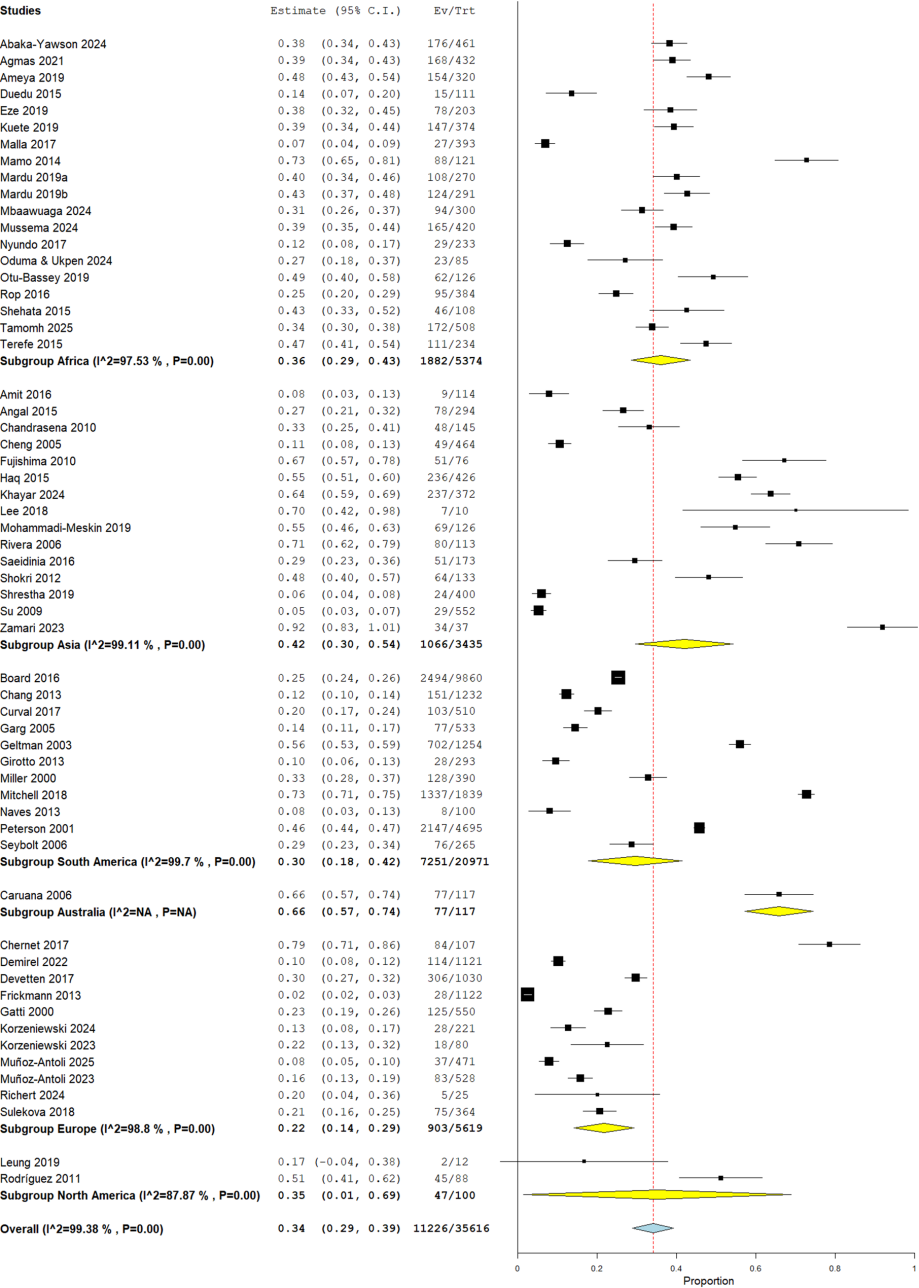
Sub-group analysis of the prevalence of IPIs based on geographical region or continent

A subgroup analysis based on geographical regions or continent was performed and revealed that Australia had the highest prevalence of intestinal parasites at 65.8% (95% CI: 57.2, 74.4), although based on a single study [28], followed by Asia with a prevalence of 42.0% (95% CI: 30.0, 54.0) which showed the widest range of prevalence rates, from as low as 7.9% (25) to a high of 91.9% [68]. Africa had a prevalence of 36.0% (95% CI: 29.0%, 43.0%), with a significant range in prevalence rates from 12.4% [56] to 48.1% [24]. The two studies from North America had a similar prevalence of 35.2% (95% CI: 0.015, 0.689), with a wide confidence interval and potential imprecision in the estimate. South America showed the second lowest prevalence of 29.6% (95% CI: 0.178, 0.415), with a relatively narrower range of prevalence rates compared to other subgroups. Europe as a subgroup had the lowest prevalence at 22.0% (95% CI: 14.0, 29.0), but also exhibited a wide range of prevalence rates from 2.5% [36] to 38.4% [45] as shown in Fig. 3.

### Sub-group analysis on the prevalence of IPIs based on the type of institutionalized population

**Fig. 4:**
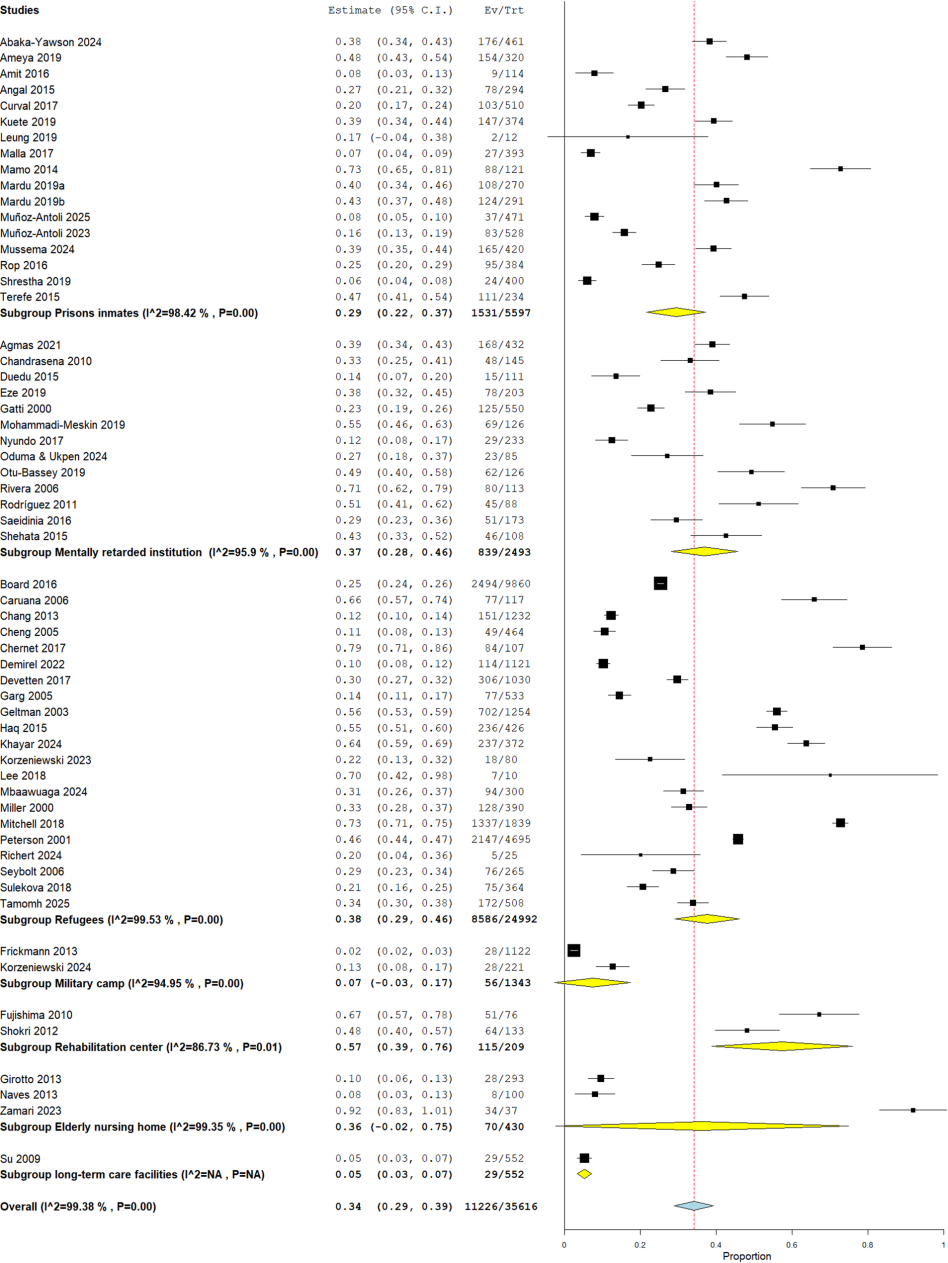
Sub-group analysis of prevalence of IPIs based on the type of institutionalized population

A subgroup meta-analysis based on the type of institutionalized population was performed to assess the prevalence of IPI. The Rehabilitation Centers had the highest prevalence of 57.3% (95% CI: 38.7%, 75.9%), but this was based on only two studies. The prevalence among individuals Psychiatric facilities followed with a prevalence of 37.0% (95% CI: 28.0%, 46.0%), with rates ranging from 12.4% [56] to 67.1% [37]. The Refugees subgroup showed prevalence of 37.5% (95% CI: 28.0%, 47.1%), similar to psychiatric facilities, with rates ranging from 10.3% [32] to 72.7% [52]. Elderly Nursing Homes had a moderate prevalence of 36.5% (95% CI: -2.4%, 74.8%), but this results is based on only two studies Prison Inmates had a slightly lower prevalence of 29.7% (95% CI: 20.8%, 38.5%), with rates ranging from a modest 6.0% [18] to 48.1% [24]. Finally, the lowest prevalence was was observed in the Military Camp subgroup and the Long-Term Care Facilities subgroup at 2.5% (95% CI: 1.6%, 3.4) [36] and 5.3% (95% CI: 3.4%, 7.1%) [66] respectively, each represented by a single study as seen in Fig. 4.

### Risk factor assessment

**Fig. 5:**
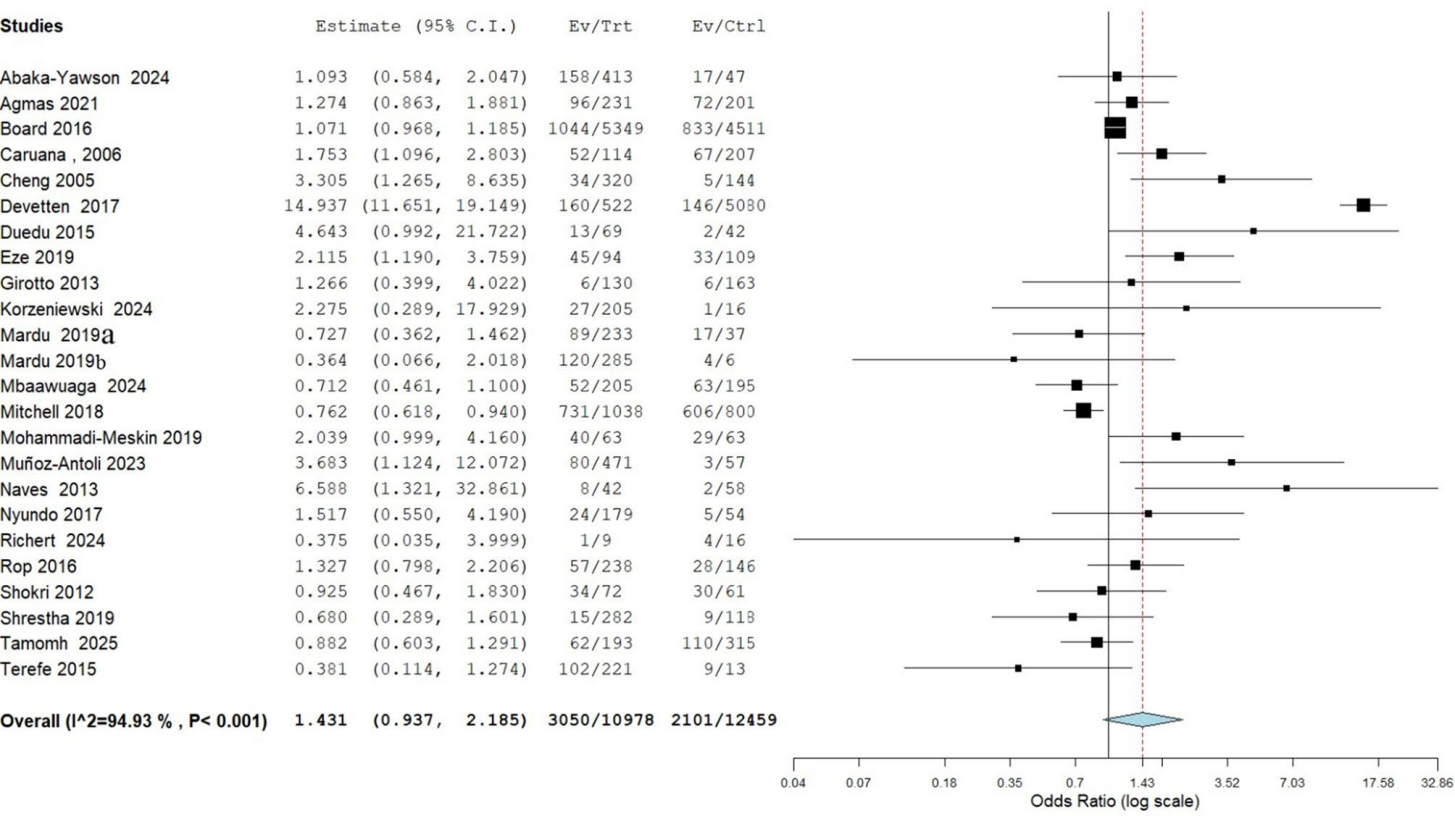
Association between gender and the occurrence of IPIs among institutionalized populations

Twenty-four studies evaluated the effect of gender on intestinal parasitic infection among various institutionalized populations. The overall odds ratio across studies was 1.43 (95% CI: 0.94–2.19), suggesting a potential increased risk associated with males compared to females, although this was not statistical significance (*p* = 0.097). However, the predisposition of males to IPIs was reported by several individual studies. For example, among the individual studies, several reported statistically significant findings. The study by De Vetten et al. [17] showed the largest effect size, with an odds ratio of 14.94 (95% CI: 11.65–19.15), indicating a substantially higher risk for males than females. Other studies with significant results favoring increased risk in males include Cheng and Wang [31] (OR 3.31, 95% CI: 1.27–8.64) and Munoz-Antoli et al. [19] (OR 3.69, 95% CI: 1.12, 12.072). In contrast, some few studies suggested a potential protective effect of being male, such as Mardu et al. [69] (OR 0.73, 95% CI: 0.36–1.46) and Mitchell et al. [52] (OR 0.76, 95% CI: 0.62, 0.94), though these results were also not statistically significant as seen in Fig. 5.

**Fig. 6:**
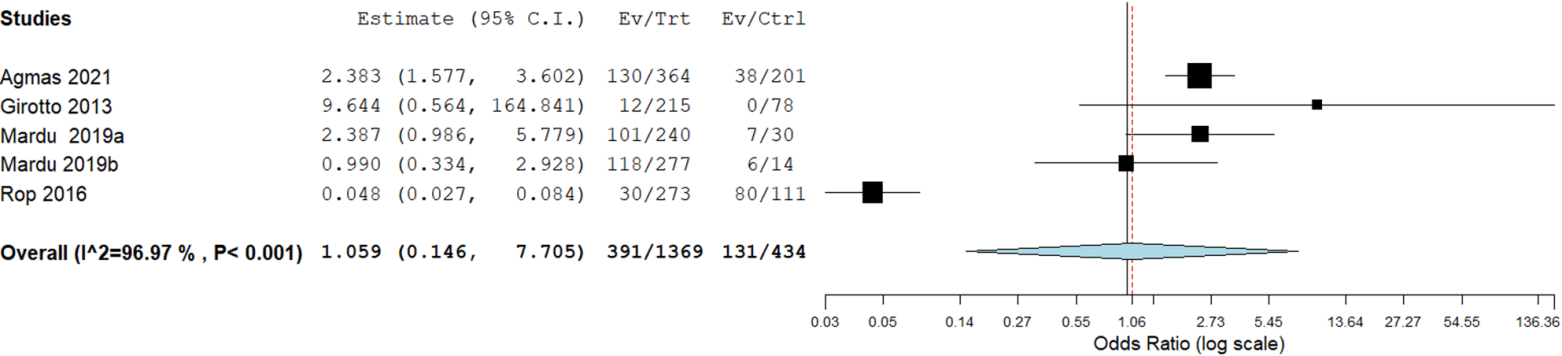
Hand washing after using toilet and its association with the occurrence of IPIs in institutionalized populations

Again, the meta-analysis assessed the effect of hand washing after using the toilet in 5 studies. The overall odds ratio across studies was 1.059 (95% CI: 0.15–7.70) and this was not statistically significant (*p* = 0.649), indicating that hand washing is not associated with intestinal parasitic infection in institutionalized populations as seen in Fig. 6.

**Fig. 7:**
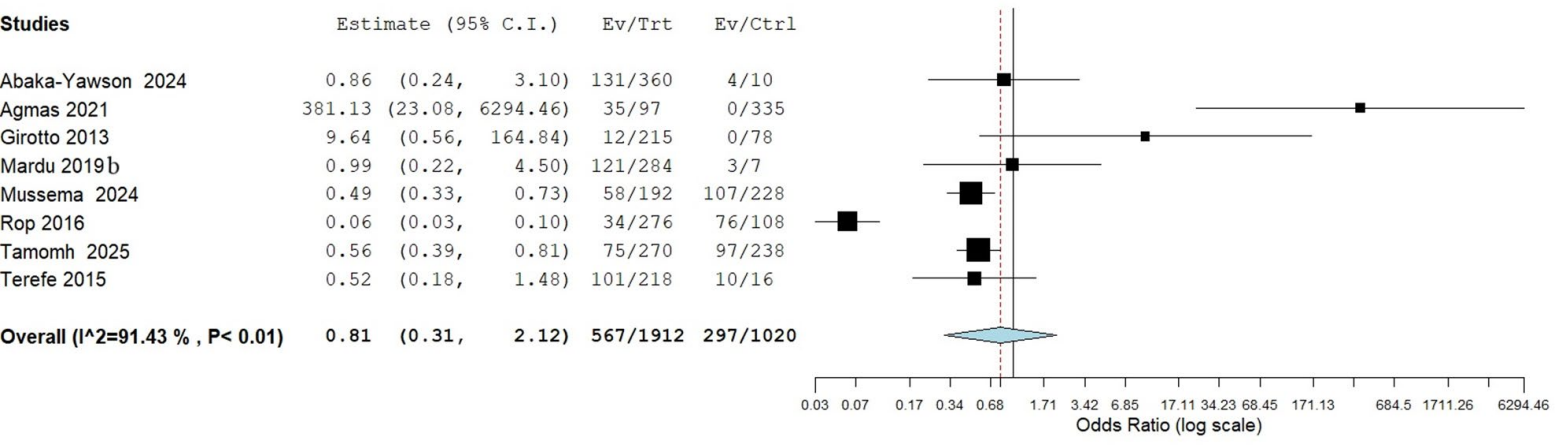
Risk association between hand washing before meals and the occurrence of intestinal parasitic infection in institutionalized populations

Similarly, the effect of hand washing before meals which involved an analysis of 8 studies also revealed that, there was no significant association between washing of hands before meals and the occurrence of intestinal parasitic infection with a pooled odds ratio of 0.81 (95% CI: 0.31–2.12, *p* = 0.670) as seen in Fig. 7.

**Fig. 8:**
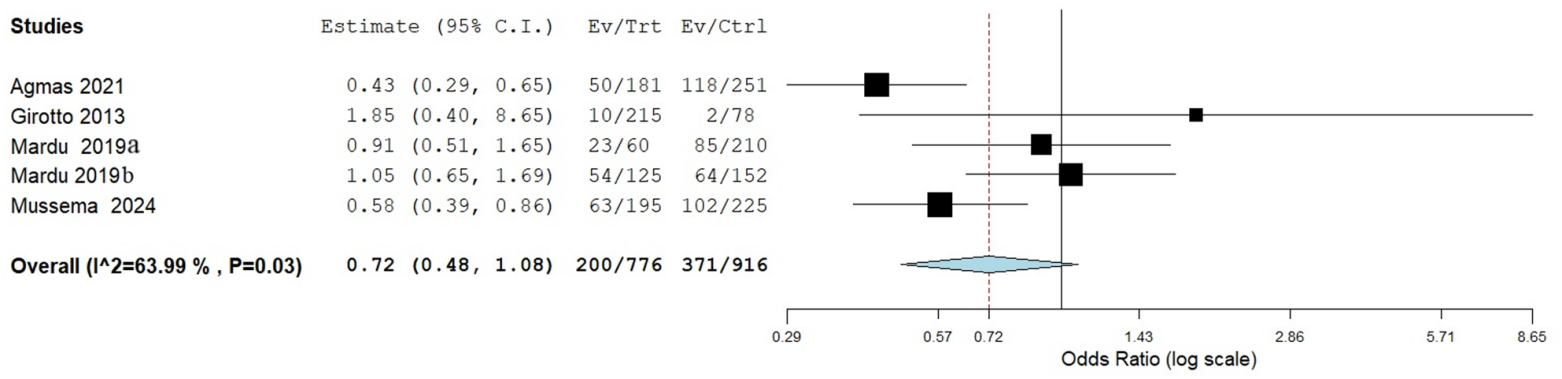
Risk association between washing hand with soap and water and the occurrence of IPIs in institutional populations compared to washing hands with water only

As seen in Fig. 8, the overall odds ratio across five studies that evaluated the effect of washing of hands with soap and water compared to washing hands with water only, and the occurrence of IPI was 0.72 (95% CI: 0.48–1.08), indicating a reduced likelihood of IPI for people who wash their hands with soap and water compared to those who wash their hands with water only, although this was not statistically significant (*p* = 0.110).

**Fig. 9:**
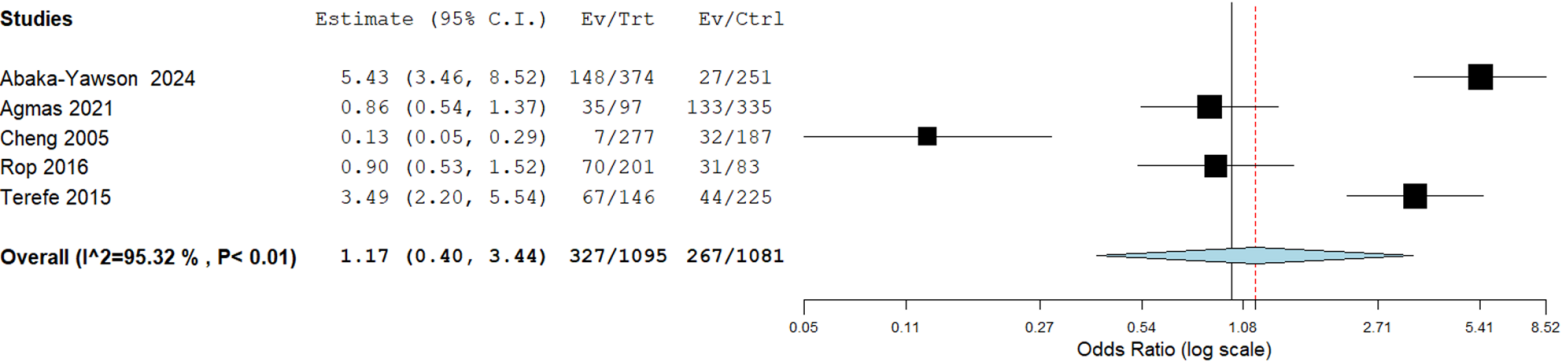
Association between literacy and the occurrence of Intestinal parasitic infections in institutional populations

Additionally, individuals with high literacy were more likely to be infected with IPI compared to non-educated or less educated ones [Pooled OR: 1.17, 95%CI: 0.29–1.16). However, this was not statistically significant (*p* = 0.770) as shown in in Fig. 9.

**Fig. 10:**
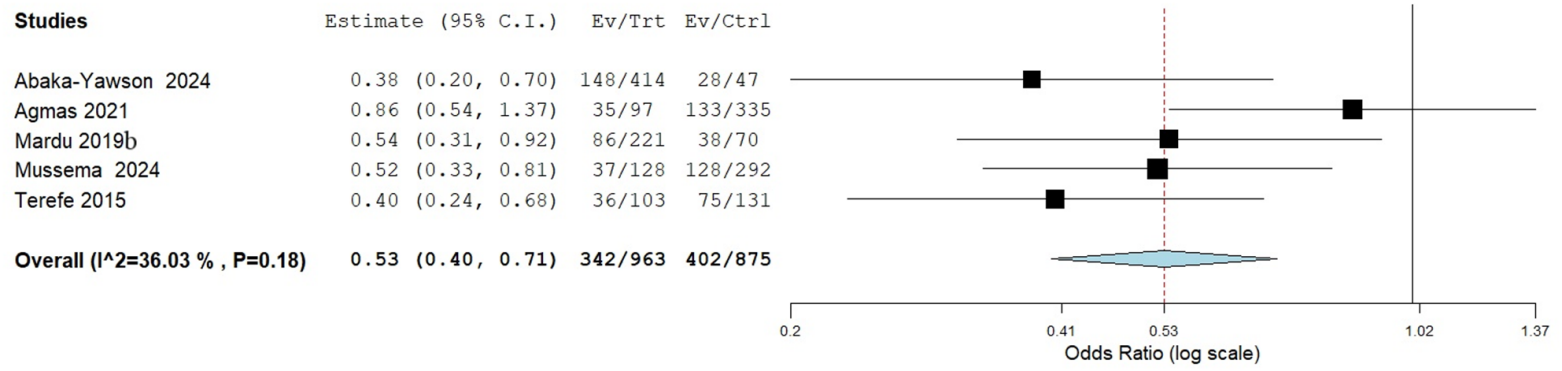
Risk association between trimming of fingernails and the occurrence of Intestinal parasitic infections in institutionalized populations compared

Furthermore, the results of five studies showed reduced of IPIs were associated with trimming of fingernails (pooled OR: 0.53, 95%CI: 0.40–3.44, *p* < 0.010) as seen in Fig. 10.

**Fig. 11:**
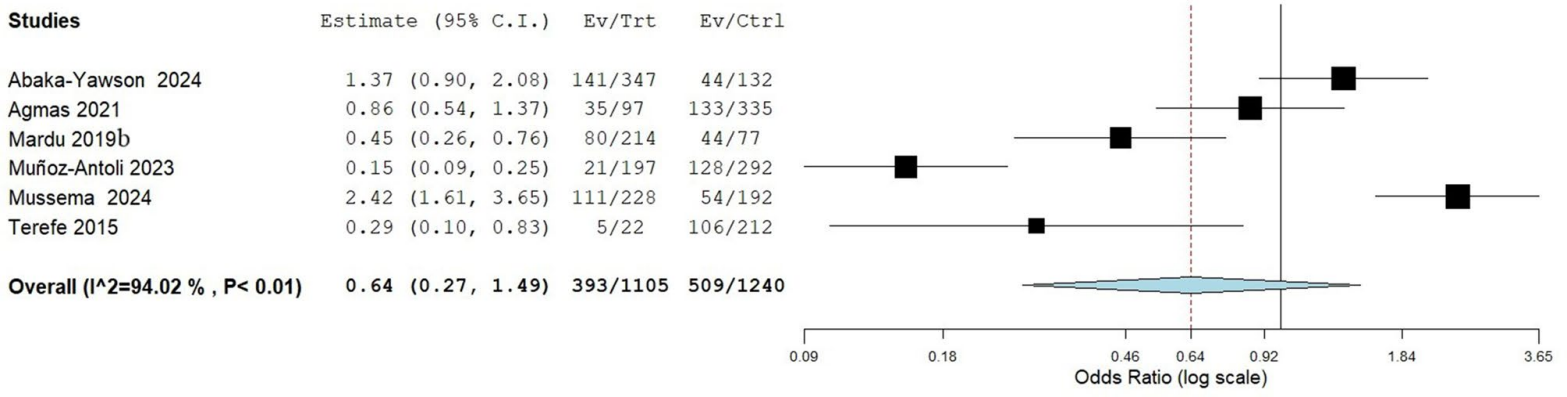
Meta-analysis of the risk association between duration of prison stay and the occurrence of IPI

It was worth noting that, the meta-analysis revealed a significant correlation between the duration of prison stay and IPI with pooled odds ratio of 2.33 (95% CI: 1.27–4.28) for individuals with shorter stay (< 3 year) compared to those with longer duration of stay as seen in Fig. 11.

### Assessment of publication bias

The Egger’s test was conducted to assess the presence of publication bias in the studies included in the systematic review. The intercept value obtained from the test was 0.38 (95%CI: -4.11 to 4.87). The p-value associated with the test was 0.8652, indicates that there is no significant evidence of publication bias in the studies included in the review, as shown in Table 3.

**Fig. 12:**
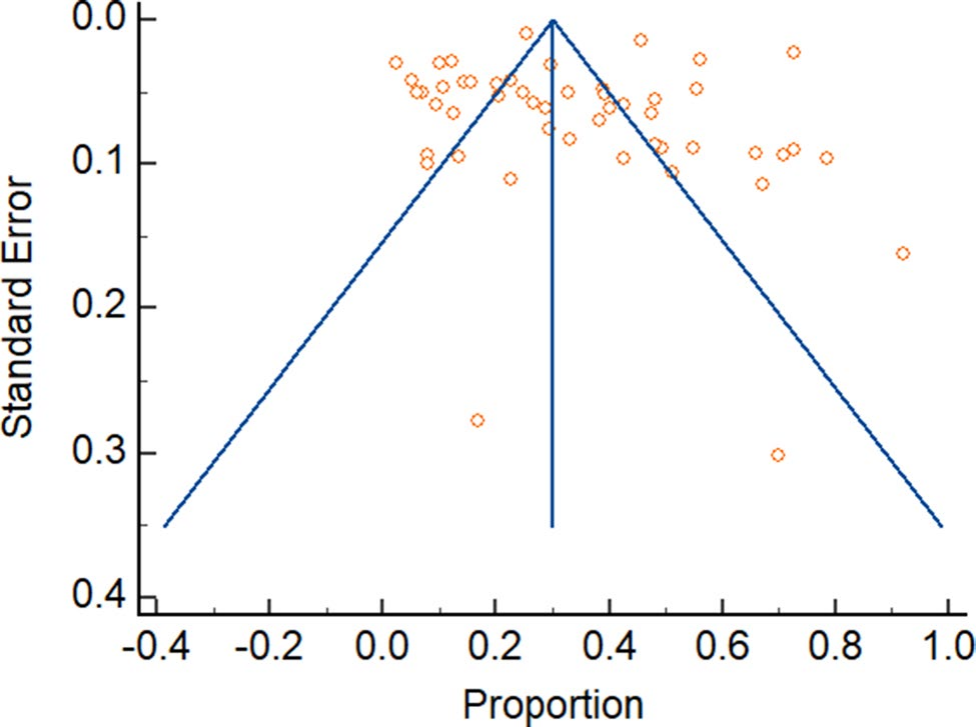
Funnel plot of studies included in the review

The funnel plots also revealed a symmetric distribution of the studies included in the review confirming of absence of publication bias as shown in Fig. 12 below.

**Table 3:** Publication bias assessment using the Egger’s test

Egger’s test	
Intercept	0.38
95% CI	-4.11 to 4.87
Significance level	*P* = 0.8652

## Discussion

Institutionalized populations such as prison inmates, psychiatric homes, refugee camps among others are characterized by overcrowding, limited access to healthcare and poor water, sanitation and hygiene (WASH) practices, creating a favourable environment for transmission of IPIs [2]. However, data on the prevalence and associated factors of IPIs among these populations remain fragmented and underreported. This study therefore sought to synthesize existing evidence to determine the overall prevalence and associated factors of IPIs among institutionalized populations to guide targeted interventions to control the infection.

First, the overall prevalence of IPIs among institutionalized populations was found to be 34.0%. Substantial heterogeneity was observed across studies (I² = 99.38%, *p* < 0.01), with 2.5% to 91.9% prevalence. Variability in sample sizes and socio-environmental characteristics had a great impact on the pooled prevalence. For instance, Leung et al. [47] used a sample size of 12 participants whilst Board and Suzuki [27] worked with 9,860 participants. This gap in sample sizes serve as a significant limitation to this study.

The pooled prevalence reported in this meta-analysis is notably lower than the prevalence of 72.7% and 70.0% as reported by Mamo [7] and Lee [8] respectively, highlighting possible disparities in environmental sanitation, access to clean water, healthcare infrastructure, and the implementation of public health interventions among the studies.

Regarding the institutionalized populations under review, individuals at rehabilitation centers have the highest prevalence of IPIs. It is important to note that individuals in rehabilitation homes are notably those recovering from substance abuse, mental challenges, and those with behavioral disorders [70]. These categories of people are at the risk of being immunocompromised due to their underlying conditions [71]. Again, individuals in rehabilitation homes are less likely to practice effective personal hygiene due to their mental health state [72]. This makes them susceptible to IPIs.

Furthermore, subgroup analysis by continent found Australia to present with the highest prevalence of IPIs. Whilst the analysis was based on a single study, it was conducted among immigrant refugees who had arrived shortly from East Africa and Cambodia [28]. With East Africa being known to contribute significantly to the global burden of IPIs [7, 12, 16] and Australia’s climatic conditions favouring few parasites, the high prevalence likely reflect imported cases rather than local transmission. On the other hand, refugees typically live in overcrowded settings with poor sanitation practices, fuelling the spread of IPIs. Other studies conducted in different continents among refugees have found prevalence to be as high as 70% in Asia [8] and 72.70% in North America [52], reflecting the widespread nature of IPIs among them.

For parasite species, this current review found *Blastocystis hominis* to be the predominant protozoan. However, other systematic reviews conducted among children of school-going age [73] as well as among inmates and street dwellers [10] have reported *Giardia lamblia* and *Entamoeba histolytica* respectively to be the most predominant protozoans.

The disparities or variations in predominance may be due to varying study populations contributing to different population characteristics. For instance, the systematic review among school-going children was limited to Ethiopia [73] whilst the study among inmate and street dwellers [10] also had most of the articles included from Africa, compared to the current review which considers articles globally.

The fact that *Ascaris lumbricoides* is the predominant helminthic infection is indeed not surprising, and consistent with findings from other systematic reviews conducted among school-going children [73] and inmates and street dwellers [10], which also reported *Ascaris lumbricoides* as the most prevalent helminth. In contrast however, a systematic review conducted among pregnant women identified Hookworm infection as the predominant IPI [74].

It is important to note that *Ascaris lumbricoides* infections have been associated with gastrointestinal disturbances such as diarrhoea and abdominal pains, as well as nutritional concerns such as weight loss, anemia and impaired growth, which should be a major concern for all stakeholders [75].

In the current review, untrimmed fingernail practices in various institutions were associated with IPIs. This section captured studies from various psychiatric and prison facilities. For prison inmates, overcrowding, limited access to potable water, and hygiene facilities leads to poor hand hygiene and ultimately leaves fingernail contaminated if left untrimmed [76]. Again, psychiatric facilities house individuals with mental health challenges which also influence their hand hygiene and fingernail trimming practices. The aforementioned characteristics of participants in these facilities increases the risk of IPIs.

## Conclusion

The pooled prevalence showed the presence of IPIs in about a third (34%) of institutionalized populations, which is a major concern. Additionally, in this review, *Ascaris lumbricoides* was the most predominant species of intestinal helminths, whereas *Blastocystis hominis* was the predominant intestinal protozoa among institutionalized populations. Further, a significant relationship was observed between fingernail trimming practices in various institutions and IPIs.

It is therefore recommended that health professionals conduct periodic screening and treatment for IPIs. Also, institutional leaders, particularly those of psychiatric and prisons facilities should consider offering nail care services to control the transmission of IPIs.

## Supplementary Information

Below is the link to the electronic supplementary material.

Supplementary Material 1

## Data Availability

All relevant data will be made available upon request from corresponding author.

## Abbreviations

ELISA: Enzyme-linked immunosorbent Assay
IPI: Intestinal Parasitic Infection
PCR: Polymerase Chain Reaction
PRISMA: Preferred Reporting Items for Systematic Review and Meta-Analysis
